# Multi-organ segmentation of abdominal structures from non-contrast and contrast enhanced CT images

**DOI:** 10.1038/s41598-022-21206-3

**Published:** 2022-11-09

**Authors:** Cenji Yu, Chidinma P. Anakwenze, Yao Zhao, Rachael M. Martin, Ethan B. Ludmir, Joshua S.Niedzielski, Asad Qureshi, Prajnan Das, Emma B. Holliday, Ann C. Raldow, Callistus M. Nguyen, Raymond P. Mumme, Tucker J. Netherton, Dong Joo Rhee, Skylar S. Gay, Jinzhong Yang, Laurence E. Court, Carlos E. Cardenas

**Affiliations:** 1 The University of Texas MD Anderson Cancer Center UTHealth Graduate School of Biomedical Sciences (GSBS), Houston, TX USA; 2 Department of Radiation Physics, The University of Texas MD Anderson Cancer Center, Houston, TX USA; 3 Department of Radiation Oncology, The University of Texas MD Anderson Cancer Center, Houston, TX USA; 4 Department of Radiation Physics, The University of Alabama at Birmingham, Birmingham, AL USA; 5 Guy’s and St, Thomas’ NHS Foundation Trust, London, UK; 6 Department of Radiation Oncology, University of California Los Angeles, Los Angeles, CA USA

**Keywords:** Translational research, Cancer, Computational science

## Abstract

Manually delineating upper abdominal organs at risk (OARs) is a time-consuming task. To develop a deep-learning-based tool for accurate and robust auto-segmentation of these OARs, forty pancreatic cancer patients with contrast-enhanced breath-hold computed tomographic (CT) images were selected. We trained a three-dimensional (3D) U-Net ensemble that automatically segments all organ contours concurrently with the self-configuring nnU-Net framework. Our tool’s performance was assessed on a held-out test set of 30 patients quantitatively. Five radiation oncologists from three different institutions assessed the performance of the tool using a 5-point Likert scale on an additional 75 randomly selected test patients. The mean (± std. dev.) Dice similarity coefficient values between the automatic segmentation and the ground truth on contrast-enhanced CT images were 0.80 ± 0.08, 0.89 ± 0.05, 0.90 ± 0.06, 0.92 ± 0.03, 0.96 ± 0.01, 0.97 ± 0.01, 0.96 ± 0.01, and 0.96 ± 0.01 for the duodenum, small bowel, large bowel, stomach, liver, spleen, right kidney, and left kidney, respectively. 89.3% (contrast-enhanced) and 85.3% (non-contrast-enhanced) of duodenum contours were scored as a 3 or above, which required only minor edits. More than 90% of the other organs’ contours were scored as a 3 or above. Our tool achieved a high level of clinical acceptability with a small training dataset and provides accurate contours for treatment planning.

## Introduction

Pancreatic cancer is one of the most aggressive tumor types, as it accounts for 3% of all cancers in the United States, as well as 7% of all cancer-related deaths^1^. Radiation therapy, along with chemotherapy, play a vital role in local tumor control for locally advanced pancreatic cancer^2^. Radiation treatment planning for pancreatic cancer is often complex with tight dose constraints^3^. This is a consequence of the pancreas being surrounded by highly radiosensitive and serial organs at risk (OARs) (duodenum, stomach, and small bowel) that require maximum dose constraints. However, OAR delineation in pancreatic and liver cancer is time consuming^4^. At our cancer center, pancreas radiation treatment requires delineation of 8 OARs: stomach, duodenum, large bowel, small bowel, liver, spleen, left kidney and right kidney. The average time spent on OAR delineation has been shown to be over 20 minutes^5^. For upper abdominal OAR delineation, reproducibility is a major challenge. Experts often have conflicting OAR delineations for the same patient, especially at the gastroesophageal junction^6^. Delineation of bowel structures (duodenum, large bowel and small bowel) is also susceptible to interobserver variability^5,7^. Margins reserved for motion management^8^ and poor soft tissue contrast at the small/large bowel border^9^ makes establishing the ground-truth for bowel structures difficult. It is often found in clinical practice that normal tissues extending (~ 1.0 cm) beyond the superior and inferior extent of the planning target volume (PTV) are not contoured on slices located outside of these margins. This is generally true for normal tissues that have a maximum dose objectives where the whole volume is not needed for dose optimization^10^, but this practice also introduces interobserver variability and clouds the establishment of the ground-truth.

Deep learning-based tools have achieved expert level performance when trained with large datasets^11–15^. It has also been shown to reduce contouring inconsistency in clinical trials and to provide more accurate dose metrics^16^. Among deep learning-driven approaches, U-Net derived models dominate in organ segmentation tasks in the abdomen^17,18^ where public datasets are abundant (liver, spleen and kidney). For serial OARs (duodenum, stomach, and small bowel) in pancreatic cancer treatment, a few U-Net based models were developed on private datasets and achieved better results than alternative approaches such as fully convolutional network-based models^19^. Wang et al. explored the multi-planar fusion approach with 2D U-Nets predicting on both axial, sagittal and coronal views^9^. Liu et al. utilized a 3D self-attention U-Net to segment the OARs in pancreatic radiotherapy^20^ and achieved state-of-the art performance. These specialized U-Net models from large academic institutions required extensive research expertise to develop. In addition, these models required at least 80 sets of complete patient contours for training and validation alone. Due to aforementioned inconsistencies in the clinical contours, extensive curation by experts is required before contours qualify for deep learning training. This expensive, time-consuming process^21^ hinders the development and adoption of deep learning models outside of large academic institutions.

Recently, the self-configuring nnU-Net framework^22^ has shown promising results in abdominal organ segmentation. This framework systematically configured U-Nets on the basis of distribution of spacings, median shape, and intensity distribution of the training CT images. The framework is also exceedingly data efficient due to robust data augmentation methods. nnU-Net has shown promising results in abdominal organ segmentation tasks and won two of the five tasks in the CHAOS challenge^18^. This framework was thus chosen as our candidate for automating upper-abdominal OAR segmentation.

In summary, upper abdominal OAR contouring is time-consuming and susceptible to variabilities. Deep learning-based auto-segmentation provides a fast and consistent alternative to manual contouring. However, specialized U-Nets and large datasets are deemed essential to a robust deep learning auto-segmentation tool according to existing literature. These requirements confine the development of auto-segmentation tool to large academic centers with research expertise. In this study, we proposed using the streamlined nnU-Net framework to customize three-dimensional (3D) U-Nets that delineate eight OARs (stomach, duodenum, large bowel, small bowel, liver, spleen, left kidney and right kidney) simultaneously on contrast-enhanced and non-contrast-enhanced CT images. We hypothesized that with a small, but consistent, training set, the standard U-Net architecture could create clinically deployable models for upper-abdominal OAR segmentation. This study demonstrated clinical utility of the automatically generated segmentations through a robust evaluation via multi-observer rating of individual contours on 75 abdominal CT scans as well as quantitative evaluation on 30 CT scans. Our approach provided an easy-to-implement, data-efficient alternative for automating the clinical workflow of pancreatic radiation treatment, including adaptive radiation therapy. Our method utilized the least amount of data to achieve clinically acceptable qualitative results and competitive quantitative results compared to existing literature. In addition, we examined the organ-by-organ segmentation performance gain as we increased the number of patients in the training dataset to provide insights on the amount of data required for training robust upper abdominal segmentation models for clinics interested in developing their own tools. We will release the entire training and testing dataset on TCIA to serve as additional resources for future abdominal organs auto-segmentation development.

## Materials and methods

### Imaging data

Seventy patients were selected from patients with pancreatic cancer who were treated at The University of Texas MD Anderson Cancer Center from 2017 to 2020 under an IRB (institutional review board) approved protocol. CT images were acquired with the breath-hold technique on Philips Brilliance Big Bore (Philips Healthcare, Best, The Netherlands) CT simulators. CT scans had pixel sizes ranged from 0.98 to 1.04 mm and slice thickness from 1 to 2.5 mm. Patients were scanned from 5 cm above the diaphragm to the iliac crest with intravenous contrast injection. The clinical OAR contours included the duodenum, small bowel, large bowel, stomach, liver, spleen, left kidney and right kidney.

### Data curation and manual segmentation

The duodenum, small bowel, and large bowel were manually delineated under physician supervision to increase consistency in normal tissue definition for these organs. To provide sufficient contextual information for the 3D U-Net models, bowel structures were extended along the z-axis and contoured throughout the entire scan. Stomach contours were trimmed to eliminate motion management margins. Liver, spleen and kidney contours were edited to ensure anatomical accuracy. All ground truth contours were reviewed and approved by a radiation oncologist. Forty sets of contours were randomly selected for training and validation. The remaining thirty sets of contours were reserved as the held-out test set.

### Data preprocessing

To segment all OARs simultaneously, labels were compiled into a single segmentation map. When organ borders overlapped, the priority of the segmentation map was duodenum, small bowel, stomach, large bowel, liver, spleen and kidneys. Organs with the most stringent dose constraints were prioritized and overwrote organs with less stringent dose constraints. All images were resampled to 0.98 mm × 0.98 mm pixel size and 2.5 mm slice thickness.

### Model training

**Figure 1:**
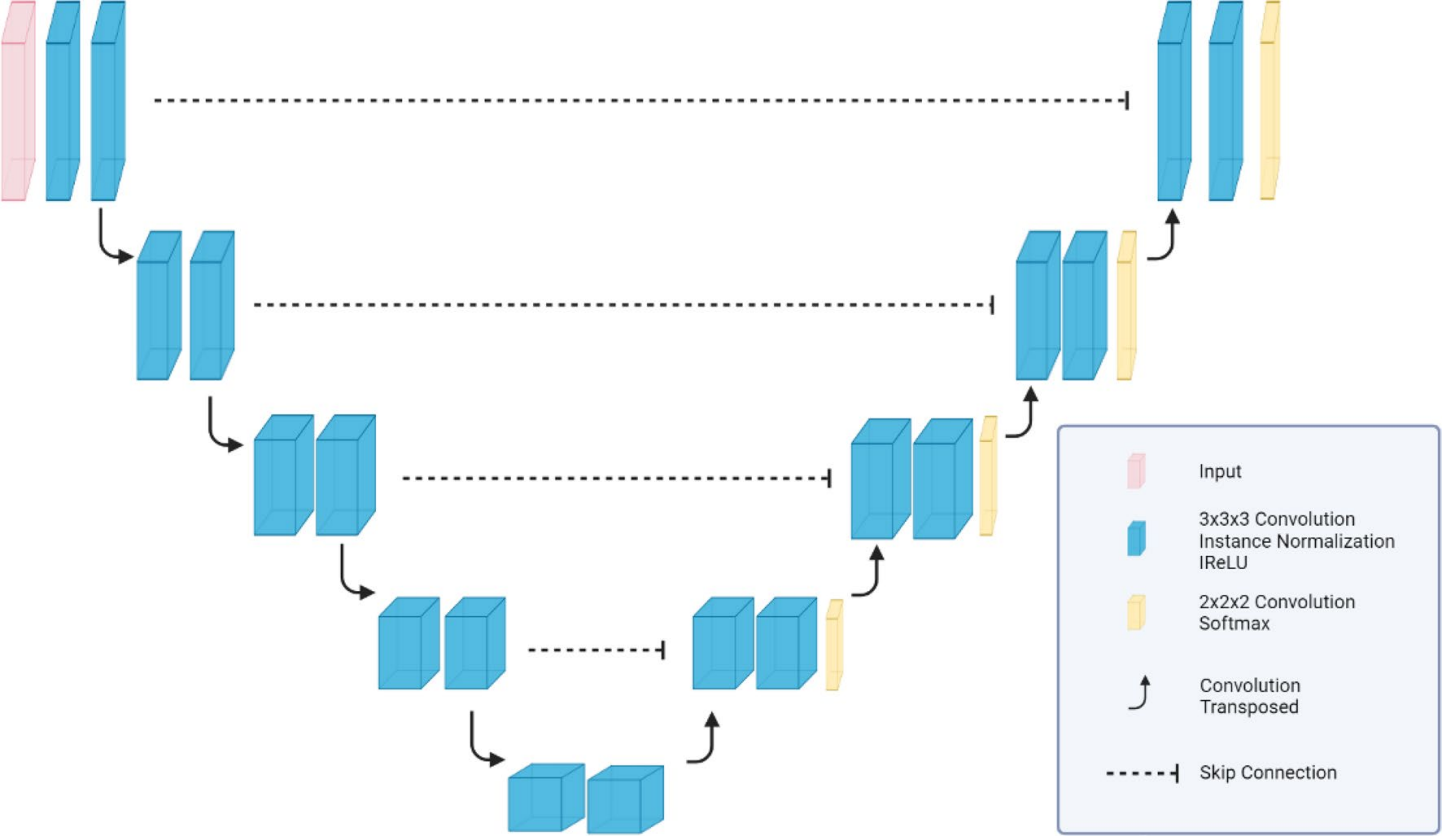
U-Net architecture customized by the nnU-Net framework based on the training dataset.

The adaptive nnU-Net framework^22^ was employed to customize 3D U-Nets for our dataset. 3D patches of image-label pairs were used for training. The patch size was 192 × 192 × 48. The 3D U-Net network depth was dynamically optimized by nnU-Net framework to ensure sufficient depth to fully utilize the large patch size. The training batch size was 2. The resulting U-Net architecture generated by the nnU-Net framework is shown in Fig. 1.

The loss function was a combination of Dice similarity coefficient (DSC) loss and cross-entropy loss. Training and testing were done on NVIDIA Tesla V100 GPUs with 32 GB VRAM. Training was stopped after 1000 epochs. To fully extract features from a small data set, five-fold cross-validation was used among the 40-patient dataset: 32 patients were used for training, and eight patients were used for validation in each fold (80-20 split). Five 3D U-Net models were trained, and the final prediction was produced by an ensemble of all five trained models from the cross validation. Training time for the U-Net ensemble was 36 hours when individual models were trained in parallel. Inference time using the U-Net ensemble for each patient was 8 minutes on average.

To evaluate performance gains as the size of training data expanded, additional model ensembles were also trained on an escalating number of patients. Subsets of 10, 15, 20, 25, 30, and 35 patients were randomly selected. The training-validation split for each set was also 80–20, which was identical to the final model ensemble. These six additional 3D U-Net ensembles were trained under the nnU-Net framework with identical training procedures.

### Quantitative evaluation

The final model ensembles from various sizes of the training data were evaluated on the held-out test set of thirty patients. The performance of the model ensembles was evaluated by the 3D DSC, 95% Hausdorff distance (HD95), and mean surface distance (MSD) between the predicted contours and the ground truth contours.

### Qualitative evaluation

An additional 75 patients simulated under the breath-hold protocol were randomly selected from the clinical database as an independent qualitative test set. Our center captures two non-contrast-enhanced and three to four contrast-enhanced CT images during simulation for patients who are suitable for CT imaging with a contrast agent. For each patient, one contrast-enhanced and one non-contrast-enhanced CT image were randomly selected as part of the qualitative analysis, resulting in a total of 150 patient CT images. The automatically generated contours on both contrast-enhanced and non-contrast-enhanced images were visually evaluated and scored using a five-point Likert scale as shown in Table 1 by five radiation oncologists from three institutions and two countries. Each image was scored once by a radiation oncologist; and each organ was scored individually.

**Table 1:** Likert scale used by physicians to evaluate contours generated on contrast- enhanced and non-contrast-enhanced CT images.

Likert scale		Explanation for this study
5	Strongly agree	Use-as-is (i.e. clinically acceptable, and could be used for treatment without change)
4	Agree	Minor edits that are not necessary. Stylistic differences, but not clinically important. The current contours/plan are acceptable
3	Neither agree or disagree	Minor edits that are necessary. Minor edits are those that the review judges can be made in less time than starting from scratch or are expected to have minimal effect on treatment outcome
2	Disagree	Major edits. This category indicates that the necessary edits are required to ensure appropriate treatment, and sufficiently significant that the user would prefer to start from scratch
1	Strongly disagree	Unusable. This category indicates that the quality of the automatically generated contours or plan are so bad that they are unusable

### Ethical approval

CT images used in this study were acquired during routine treatment. This study was approved by the University of Texas MD Anderson Cancer Center Institutional Review Board (IRB 4), which included a waiver of informed consent, and all methods were performed in accordance with the relevant guidelines and regulations.

## Results

### Quantitative evaluation

A summary of the quantitative evaluation (n = 30) is provided in Table 2. All automatically generated contours had a mean DSC value of 0.80 or higher when compared to the ground-truth contours. Solid organs such as liver, spleen and kidneys all achieved mean DSC values ranging from 0.96 to 0.97. Radiosensitive hollow organs such as small bowel, large bowel and stomach achieved mean DSC values ranging from 0.89 to 0.92. Duodenum achieved a mean DSC of 0.80. For distance metrics, solid organs (liver, spleen and kidneys) had mean HD95 ranging from 2.21 to 2.51 mm and mean MSD ranging from 0.61 to 1.07 mm. Radiosensitive hollow organs (small bowel, large bowel and stomach) had mean HD95 ranging from 4.77 to 7.77 mm and mean MSD ranging from 1.23 to 1.99 mm. Duodenum had a mean HD95 of 12.34 mm and mean MSD of 1.68 mm.

**Table 2:** Mean Dice similarity coefficient (DSC), 95% Hausdorff distance (HD95), and mean surface distance (MSD) between ground truth and prediction results from our tool on contrast-enhanced CT images.

	DSC		HD95 (mm)		MSD (mm)	
	Mean	SD	Mean	SD	Mean	SD
Duodenum	0.80	0.08	12.34	9.09	1.68	1.04
Small bowel	0.89	0.05	7.77	8.90	1.99	2.10
Large bowel	0.90	0.06	7.15	8.42	1.27	0.87
Stomach	0.92	0.03	4.77	2.98	1.23	0.78
Liver	0.96	0.01	3.56	1.71	1.07	0.49
Spleen	0.97	0.01	2.21	1.27	0.56	0.23
Kidney left	0.96	0.01	2.51	1.29	0.59	0.18
Kidney right	0.96	0.01	2.52	0.90	0.61	0.19

**Figure 2:**
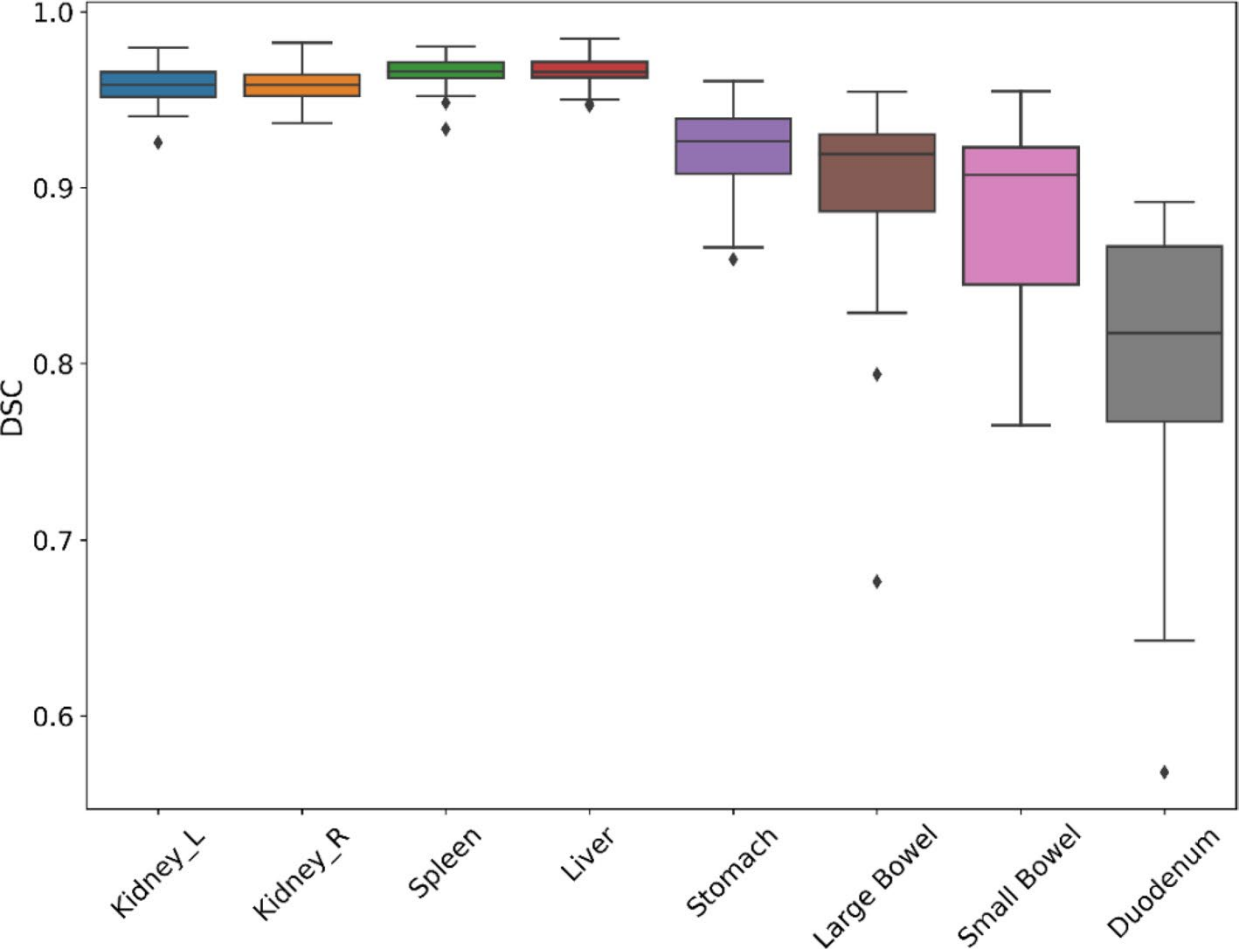
Box and whisker plots of Dice similarity coefficient (DSC) distance between ground-truth and automatically generated contours by our tool on contrast-enhanced CT images. The central line represents the median value. The border of the box represents the 25th and 75th percentiles. The outliers are represented by diamond markers.

DSC boxplots of all organs were shown in Fig. 2. Auto-segmentation performance had more variability in hollow organs compared to solid organs. Outliers from small bowel and large bowel auto-segmentations were often caused by misidentification of small/large bowel in inferior regions of CT scans outside of treatment fields. Low DSC examples of duodenum were often caused by disagreements at the stomach/duodenum and duodenum/jejunum borders.

**Figure 3:**
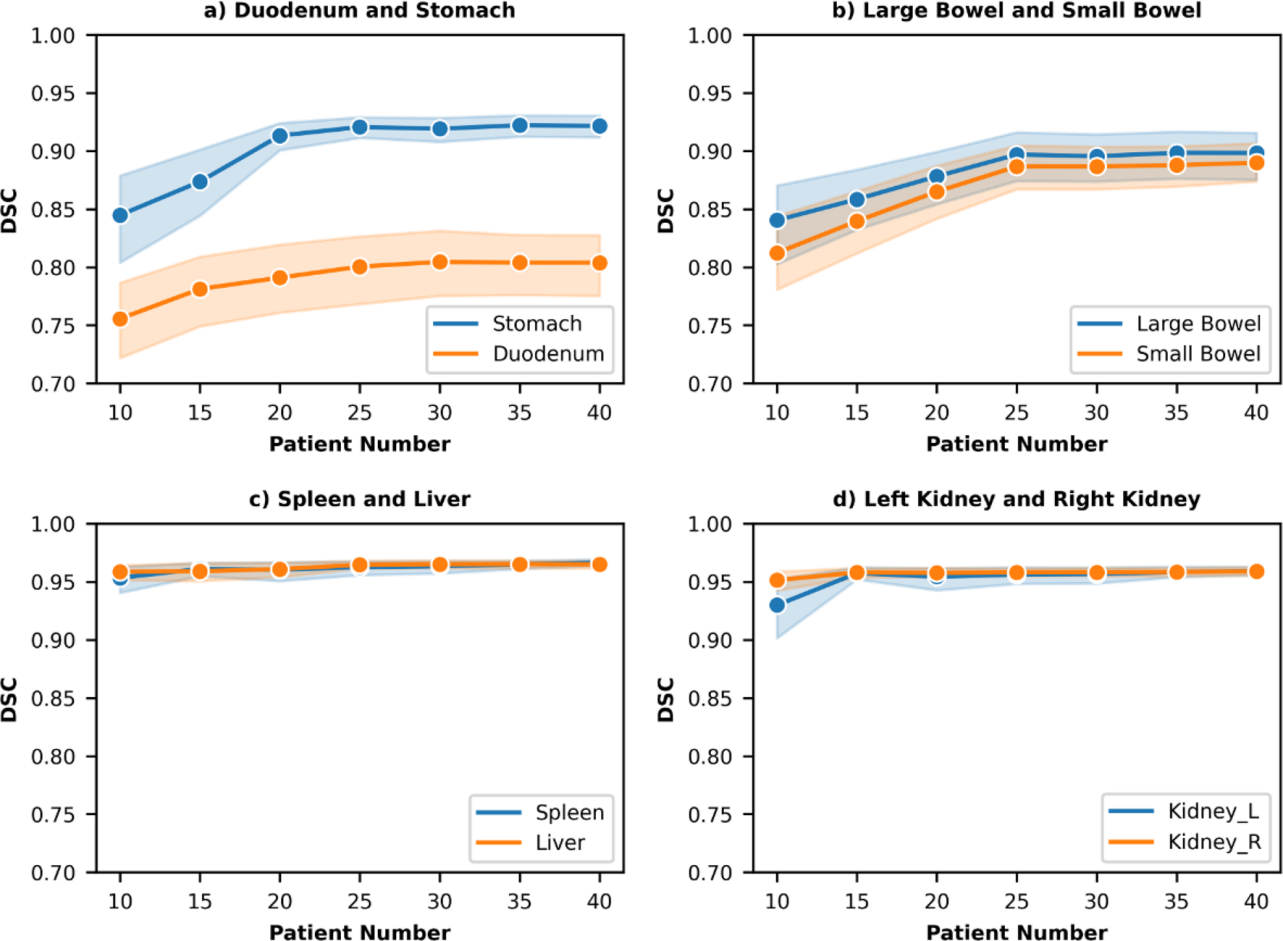
Mean DSC values between automatically generated contours and ground- truth contours increased as the number of patients in the dataset increased. The shadow represents the corresponding standard deviation for individual DSC values.

In order to determine if 40 patients were sufficient for optimal model performance, the mean DSCs for the individual organs were also examined for an escalating number of patients. The result was plotted in Fig. 3. The mean DSC increased as the size of the training dataset increased. The mean DSCs of all organs tended to converge as the number of patients approached 40.

### Qualitative evaluation

The results from physicians’ qualitative evaluations are shown below in Tables 3. Among the non-contrast-enhanced CT images, 85.3% of the duodenum contours, 92.0% of the small bowel contours, 93.3% of the stomach contours and more than 95% of the other organ contours received a score of 3 or greater, suggesting that these contours required only minor edits from physicians. More than 50% of the duodenum, small bowel, large bowel, and stomach contours as well as more than 85% of the spleen and kidney received a score of 4 or above.

**Table 3:** Qualitative scores for contours generated on contrast-enhanced and non- contrast-enhanced CT images of 75 randomly selected patients.

	Non-contrast-enhanced CT Images				Contrast-enhanced CT Images			
	< 3	≥ 3	≥ 4	5	< 3	≥ 3	≥ 4	5
Duodenum	14.7%	85.3%	50.7%	18.0%	10.7%	89.3%	60.0%	22.0%
Small bowel	8.0%	92.0%	58.7%	28.0%	5.3%	94.7%	62.7%	30.0%
Large bowel	2.7%	97.3%	62.7%	28.0%	2.7%	97.3%	69.3%	30.0%
Stomach	6.7%	93.3%	62.7%	38.0%	4.0%	96.0%	66.7%	38.0%
Liver	4.0%	96.0%	77.3%	60.0%	2.7%	97.3%	84.0%	66.0%
Spleen	1.3%	98.7%	90.7%	86.0%	1.3%	98.7%	93.3%	86.0%
Kidney left	1.3%	98.7%	90.7%	70.0%	1.3%	98.7%	94.7%	74.0%
Kidney right	2.7%	97.3%	86.7%	66.0%	1.3%	98.7%	93.3%	72.0%

**Figure 4:**
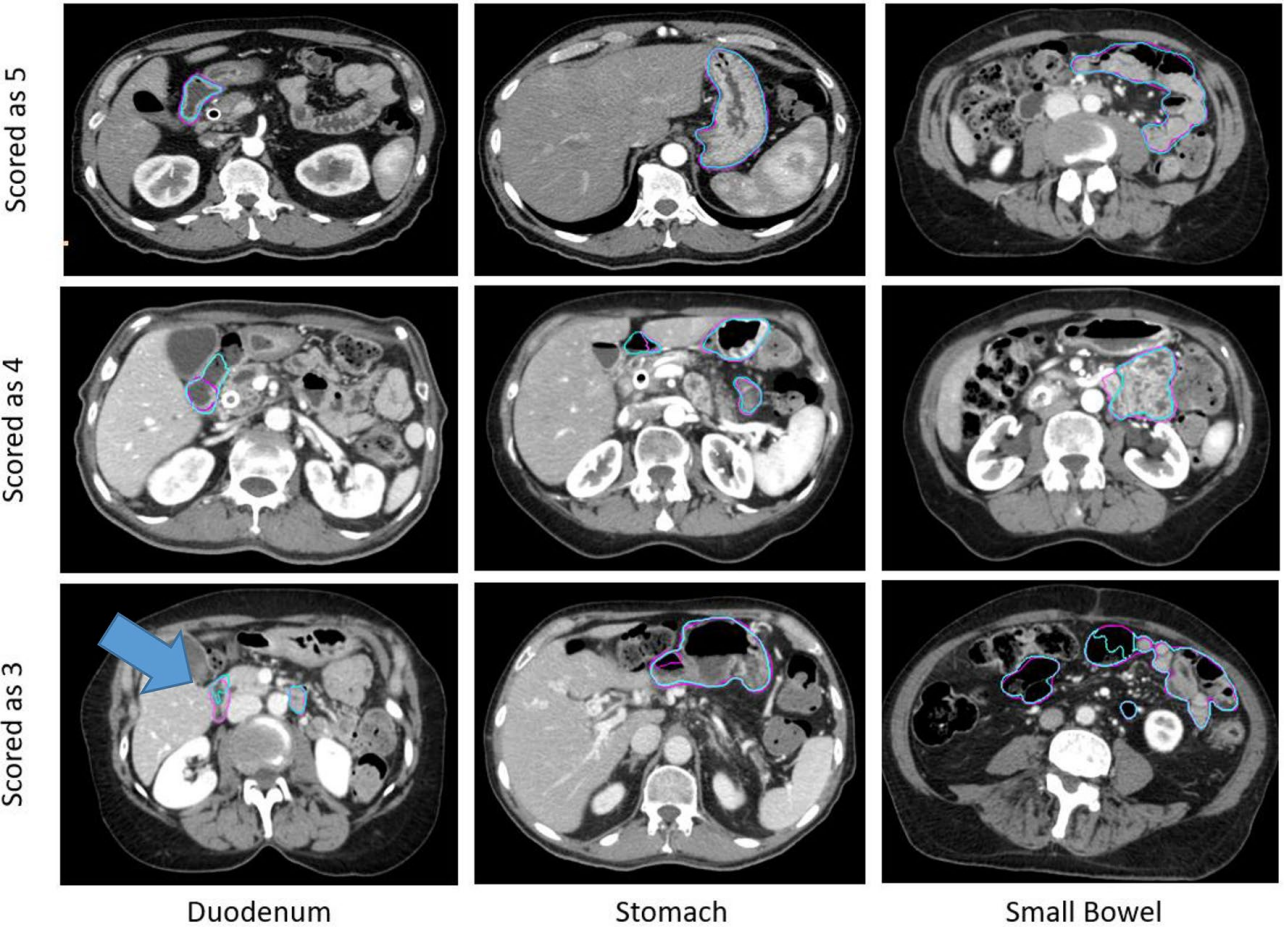
Representative contours of organs scored on a Likert scale as 5, 4, and 3 (top to bottom) by physicians. The ground truth contours are shown as purple in all images. The automatically generated contours are shown as cyan in all images. The arrow indicated a segment of under-contoured duodenum that required minor edits.

There was a small improvement in contour scores for auto-segmentations on contrast-enhanced CTs. 89.3% of the duodenum contours, 94.7% of the small bowel contours, and more than 95% of the other organ contours were scored as a 3 or greater. More than 60% of the duodenum, small bowel, large bowel, and stomach contours and more than 90% of the spleen and kidney scored a 4 or greater. Examples of automatically generated contours scored as 3,4 and 5 for duodenum, stomach and small bowel are shown in Fig. 4.

## Discussion

We have developed a deep-learning-based tool for accurate and robust upper-abdominal OAR auto-segmentation. Our tool could simultaneously segment the duodenum, large bowel, small bowel, stomach, liver, spleen, and kidneys. Upon evaluation, the tool performed well in both quantitative and qualitative assessments. These tests were conducted on randomly selected held-out test patients (30 and 75 patients for quantitative and qualitative assessments, respectively). Our qualitative assessment was conducted by five radiation oncologists from three different institutions. The tool achieved acceptable performance for clinical deployment, even though it was trained and validated with only 40 patients. Based on the results from this study, we have clinically implemented this auto-contouring system in the clinic at MD Anderson Cancer Center. In the future, we will make this auto-contouring tool available as part of the Radiation Planning Assistant^23^ (rpa.mdanderson.org) to make this tool available to radiation oncology clinics in low- and middle-income countries.

Deep learning-based auto-segmentation approaches typically require a large amount of high-quality segmented datasets to achieve optimal performance^24^. In clinical scenario, the amount of high-quality labeled images is limited^25^. Creating high-quality contours suitable for deep learning training requires significant time resources and expertise^21,26^. A number of self-supervised deep learning approach were proposed by generating artificial data^27–29^, but these approaches required technical expertise only available at large academic centers. Our findings offered an affordable, easy to implement approach to create auto-segmentation tools when public dataset is not available. The self-adaptive nnU-Net framework provided a standardized platform for U-Net architectures, allowing us to customize 3D U-Net ensembles that maximized the performance of the U-Net architecture. The qualitative evaluation provides evidence for the prowess of our tool. Automatically generated contours received a Likert score of 3 or above required only minor edits. Physicians deemed these contours beneficial to their segmentation workflow. Among 75 independent test patients, over 90% of the automatically generated contours received a Likert score of 3 or greater on most organs. For organs with poor soft tissue boundaries such as the duodenum, 89.7% of CT contours only required minor edits for clinical use. Our results have shown that with a dataset of 40 patients, a standard 3D U-Net architecture could deliver automatically generated contours suitable for clinical deployment.

**Figure 5:**
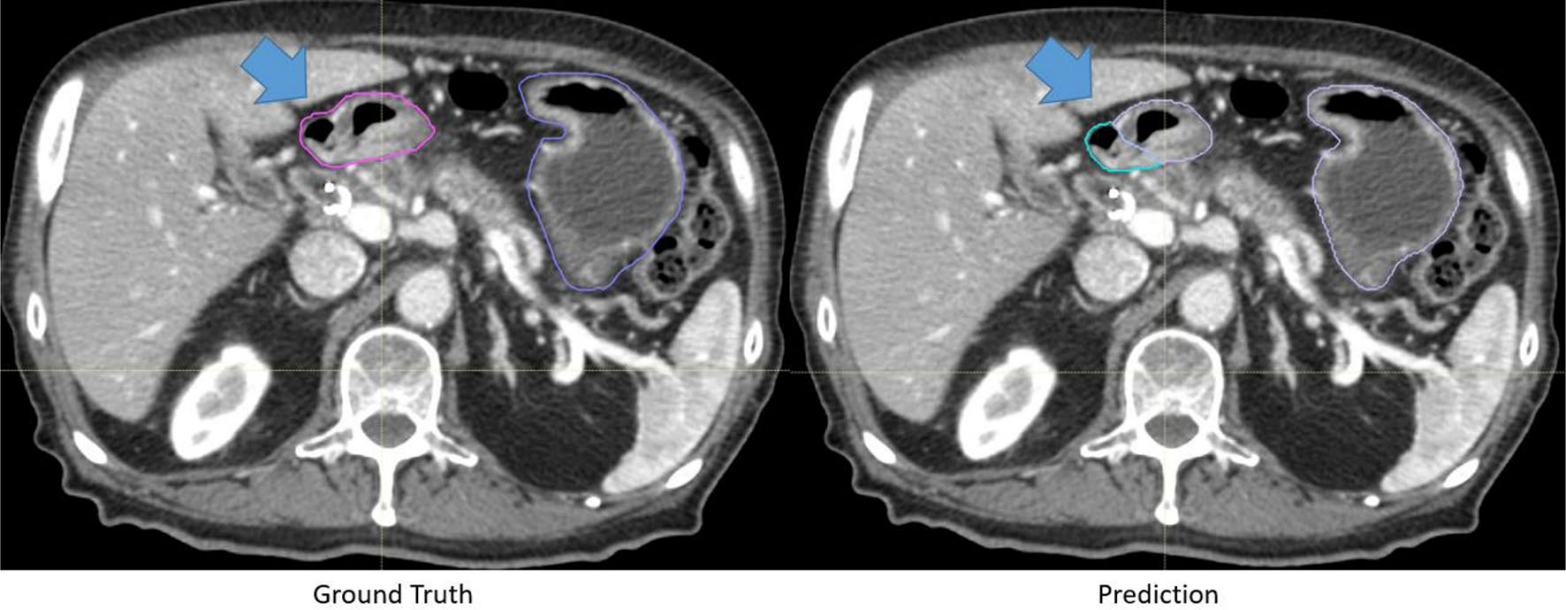
Representative ground-truth (left) and the automatically generated (right) contour of a patient’s duodenum and stomach. These contours differed significantly, but because the duodenum and stomach are often optimized using the same dose constraints (i.e. Dmax < 28 Gy), the contours were scored as a 4 and deemed acceptable for treatment planning.

Clinical context of segmentation errors differentiated acceptable contours (Likert ≥ 4) from contours needed necessary minor edits (Likert = 3). Small contour errors may have significant clinical relevancy. For the duodenum contour scored as a 3 in Fig. 4, the tool under-contoured a portion of the duodenum as shown by the arrow. The error shown was critical to patient safety because this segment of the duodenum was medially located and was close to the treatment target. Although most of the duodenum was properly contoured, the generated contour was scored as a 3 instead of a 4. The edit required from physicians, however, was marginal. Physicians were less concerned about absolute anatomical accuracy in other cases. For example, interobserver variability could be significant at the border of stomach and duodenum. The anatomical landmarks used to distinguish the two are subtle, often lacking a clear border. While the generated contour deviated drastically from the ground truth as shown in Fig. 5, it was scored as a 4 and deemed acceptable for treatment planning by physicians. This was because the duodenum and stomach are often optimized to have the same maximum dose constraints (D_max_ < 28 Gy).

**Figure 6:**
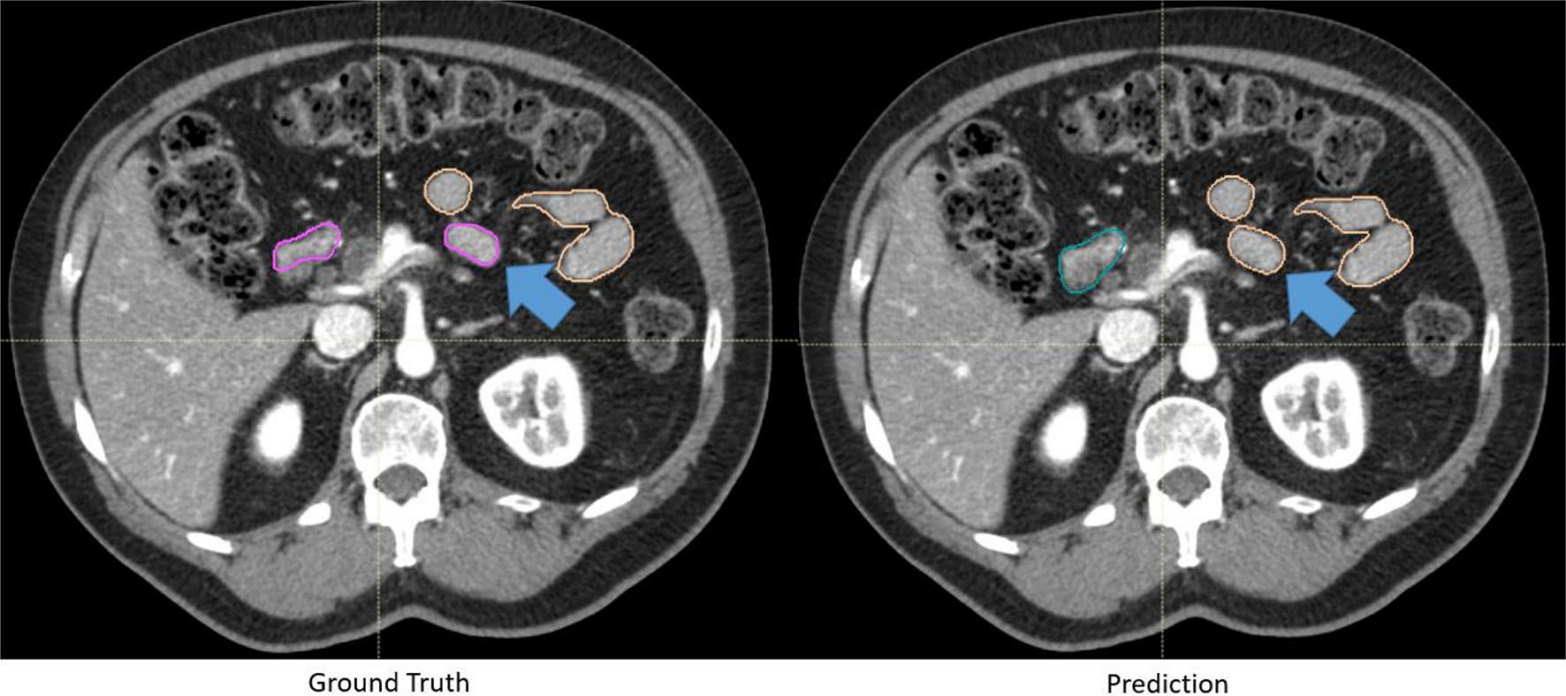
Representative ground truth (left) and the automatically generated (right) contours of a patient’s duodenum and small bowel (jejunum). The ground truth is ambiguous at the transition from duodenum to small bowel (jejunum). The deviation from the ground truth was deemed as a stylistic difference.

Individual stylistic preferences differentiated use-as-is contours (Likert = 5) from the acceptable contours (Likert = 4). These stylistic preferences were the most prominent at the intersection of the duodenum and jejunum (contoured as part of the small bowel). The superior border of the fourth section of the duodenum had no visible border features on CT images. In Fig. 6, the automatically generated contour was scored as a 4. The ground truth duodenum contour extended more superiorly compared to the automatically generated contour at the region indicated by the arrow. The varying cranial ends of duodenum contours were deemed as stylistic differences. The physicians were uncertain about the anatomical ground truth in the region. Since duodenum and small bowel were often optimized to have the same maximum dose constraints (D_max_ < 28 Gy), physicians decided that these differences had limited impact on treatment planning.

Our quantitative results are comparable to those of state-of-the-art models trained with datasets of 80 patients or more for most organs. The DSC scores of the tool on small bowel, large bowel, stomach, spleen, liver, and kidney contours were within 0.01 of the current 3D state-of-the-art model (Liu et al.) as shown in Table 4. The MSDs were also comparable or smaller than the 3D state-of-the-art model shown in Table 5. Our tool, however, was trained and validated with a much smaller dataset of 40 patients. Our approach seemed to be more data efficient compared to the state-of-the-art approach. As data curation process is known to be time-consuming and expensive, our method would allow easier development and adoption in the clinic.

**Table 4:** Dice similarity coefficient comparison between our tool and other state-of-the- art upper-abdominal auto-segmentation models.

	Ours (n = 40)		Liu et al. (n = 80)		Wang et al. (n = 177)	
	Mean	SD	Mean	SD	Mean	SD
Duodenum	0.80	0.08	0.86	0.06	0.75	9.10
Small Bowel	0.89	0.05	0.89	0.06	0.80	10.20
Large Bowel	0.90	0.06	0.91	0.03	0.83	7.40
Stomach	0.92	0.03	0.93	0.03	0.95	2.60
Liver	0.96	0.01	0.96	0.01	0.98	0.70
Spleen	0.97	0.01	NA	NA	0.97	1.50
Kidney Right	0.96	0.01	0.95	0.02	0.98	2.10
Kidney Left	0.96	0.01	0.95	0.02	0.97	1.90

**Table 5:** Mean surface distance comparisons between our tool and other state-of-the-art upper-abdominal auto-segmentation models.

	Ours (n = 40)		Liu et al. (n = 80)		Wang et al. (n = 177)	
	Mean (mm)	SD (mm)	Mean (mm)	SD (mm)	Mean (mm)	SD (mm)
Duodenum	1.68	1.04	1.39	0.54	1.36	1.31
Small Bowel	1.99	2.10	1.99	1.08	3.01	3.35
Large Bowel	1.27	0.87	1.67	0.55	3.59	4.17
Stomach	1.23	0.78	1.77	1.19	1.68	1.55
Liver	1.07	0.49	1.45	0.80	1.23	1.52
Spleen	0.56	0.23	NA	NA	0.42	0.25
Kidney Right	0.59	0.18	1.05	0.86	0.45	0.89
Kidney Left	0.61	0.19	1.06	0.79	0.30	0.30

Studies have suggested that 3D models demand too many parameters and required a large training dataset^30^ to converge. Previous state-of-the-art approaches, such as organ-attention 2D deep networks with reverse connections by Wang et al., have been developed to segment 2D slices along axial, sagittal, and coronal views to reduce the number of trainable parameters^9^. Our tool outperformed the 2D-based multi-planar fusion approach in DSC for duodenum, small bowel and large bowel as shown in Table 4. We also achieved lower MSD for small bowel, large bowel, stomach and liver as shown in Table 5. When challenged with structures that span along the z-axis, 3D models were better equipped to segment these structures compared to 2D-based multi-planar fusion model due to its capability to capture anatomical context along the z-axis. Since only 40 patients were used for training and validation, our tool’s 3D approach seemed to be more data efficient than the 2D multi-planar fusion approach as well.

The model performance progression with increasing patient number (Fig. 3) gave us a better perspective on why our quantitative results were comparable to state-of-the-art models. For challenging hollow structures such as the stomach and duodenum, the 3D U-Net models initially gained performance as the patient number increased. The DSC curve started converging as we approached 25 patients. Similar trends were observed in the large bowel and small bowel DSCs. While the mean DSCs converged, the standard deviations were decreasing for the stomach, large bowel and small bowel. Prediction results were less variable with a larger training/validation dataset. For solid organs such as the spleen, liver, and kidney, DSC scores were above 90 even with only 10 patients. This data provides insights for clinics or individuals that are interested in developing their individual 3D U-Net models for upper-abdominal organ segmentation. When faced with the task of creating auto-segmentation tools with a limited annotation budget, our findings might be a guideline for budget allocation.

Our tool was developed and tested on the ground truth label delineated according to our institution’s implementation of the RTOG guideline. While we introduced five radiation oncologists from three institutions to conduct qualitative evaluation, the test patients were from the same institution. With varying imaging protocols, image acquisition and reconstruction parameters, the model performance might suffer if the test patients were from various institutions from our experience^31^. In this case, small training samples might not be sufficient to guarantee great performance across varying patient cohorts. Further evaluation is needed to assess the model ensemble’s performance on different patient populations.

For future work, automatic quality assurance of the generated contour, i.e. capturing clinically unusable contours, would also be a crucial addition to our automation tool. In addition, our center utilizes CT-on-rails image guided system for pancreatic radiation treatment. While our tool exhibited robust qualitative results on non-contrast-enhanced CT images, future work would include dose accumulation studies using automatically generated contours. This can pave the way for adaptive radiation therapy in pancreatic radiation treatment.

## Conclusions

We proposed a simple but effective approach for developing a deep learning-based segmentation model for upper-abdominal OAR segmentation. Using only 40 patients, we trained a nnU-Net model to generate automatic contours that was able to produce clinically acceptable results on both contrast-enhanced and non-contrast-enhanced CT images. The results of the presented analysis led to the clinical deployment of this tool.

## Data Availability

The data can be made available on request to Laurence Court (lecourt@mdanderson.org). The dataset will be available on The Cancer Imaging Archive.
